# Virus-like Particle Vaccine Expressing the Respiratory Syncytial Virus Pre-Fusion and G Proteins Confers Protection against RSV Challenge Infection

**DOI:** 10.3390/pharmaceutics15030782

**Published:** 2023-02-27

**Authors:** Su-Hwa Lee, Ki-Back Chu, Min-Ju Kim, Jie Mao, Gi-Deok Eom, Keon-Woong Yoon, Md Atique Ahmed, Fu-Shi Quan

**Affiliations:** 1 Department of Medical Zoology, Kyung Hee University School of Medicine, Seoul 02447, Republic of Korea; 2 Medical Research Center for Bioreaction to Reactive Oxygen Species and Biomedical Science Institute, Core Research Institute (CRI), Kyung Hee University, Seoul 02447, Republic of Korea; 3 Department of Biomedical Science, Graduate School, Kyung Hee University, Seoul 02447, Republic of Korea; 4 ICMR-Regional Medical Research Centre, NE Region, Dibrugarh 786010, India

**Keywords:** respiratory syncytial virus, virus-like particle, pre-fusion, glycoprotein, vaccine

## Abstract

Respiratory syncytial virus (RSV) causes severe lower respiratory tract disease in children and the elderly. However, there are no effective antiviral drugs or licensed vaccines available for RSV infection. Here, RSV virus-like particle (VLP) vaccines expressing Pre-F, G, or Pre-F and G proteins on the surface of influenza virus matrix protein 1 (M1) were produced using the baculovirus expression system, and their protective efficacy was evaluated in mice. The morphology and successful assembly of VLPs were confirmed by transmission electron microscope (TEM) and Western blot. High levels of serum IgG antibody response were detected in VLP-immunized mice, and significantly higher levels of IgG2a and IgG2b were found in the Pre-F+G VLP immunization group compared to the unimmunized control. Serum-neutralizing activity was higher in the VLP immunization groups compared to the naïve group, with Pre-F+G VLPs demonstrating superior neutralizing activity to the single antigen-expressing VLP groups. Pulmonary IgA and IgG responses were generally comparable across the immunization groups, with VLPs expressing the Pre-F antigen eliciting higher IFN-γ in spleens. The frequencies of eosinophils and IL-4-producing CD4^+^ T cell populations were substantially lower in the lungs of VLP-immunized mice, with the PreF+G vaccine inducing a significant increase in CD4^+^ and CD8^+^ T cells. VLP immunization significantly decreased the viral titer and inflammation in the lungs of mice, with Pre-F+G VLPs conferring the best protection. In conclusion, our present study suggests that the Pre-F+G VLPs could be a potential vaccine candidate against RSV infection.

## 1. Introduction

Respiratory syncytial virus (RSV) is a single-stranded RNA virus belonging to the Family *Pneumoviridae* under the genus *Orthopenumovirus*, which can be further subdivided into antigenic groups A and B [1]. The virus was first discovered in 1956 and has been pointed out as the most important cause of lower respiratory tract disease in infants under 1 year of age worldwide [2,3,4]. Globally, it is estimated that there are over 33 million cases of RSV-related infection each year in children under 60 months of age, with an estimated 101,400 deaths from RSV infection in 2019 [5,6]. Additionally, in a study of children under 59 months of age who were hospitalized for severe pneumonia in seven developing countries, it was reported that 31% of children had RSV-induced pneumonia [7]. In the 1960s, the development of vaccine-enhanced disease (VED) in two children who were immunized with the formalin-inactivated RSV (FI-RSV) vaccines resulted in their deaths [8,9]. As a consequence of this incident, RSV vaccine development has progressed rather cautiously, and developing a vaccine that confers a durable virus-neutralizing response whilst avoiding VED induction remains a monumental hurdle.

Fusion protein (F) and attachment glycoprotein (G) are the two major surface antigens of RSV that are frequently utilized in vaccine development. Numerous studies over decades have identified several factors in these antigens which can affect immunogenicity or VED induction. For example, it was recently identified that vaccines based on the pre-fusion (Pre-F) conformation of the F antigen are more immunogenic and provide a greater breadth of protection than the post-fusion form [10,11]. The Pre-F conformation is highly conserved among RSV isolates and possesses highly neutralization-sensitive antigenic sites that are not present in the post-fusion form of the protein, thus signifying its developmental potential as a major candidate antigen for RSV vaccines [12,13,14]. Considerable interest has also been placed on RSV G proteins. Vaccines expressing the RSV G protein elicited neutralizing antibody responses while suppressing the formation of the RSV-induced pulmonary pathology in immunized mice [15]. Antibodies induced by the G protein, which are known to have immunomodulatory effects, can induce some neutralizing activity and have been reported to reduce toxicity and improve immune responses during subsequent infection [16,17,18]. For these reasons, a growing number of studies are investigating the efficacy elicited by vaccines expressing both Pre-F and G antigens.

Virus-like particles (VLPs) are highly immunogenic nanoparticles that mimic the native virion structure and express multiple copies of antigenic proteins on their surface while being incapable of replicating in host cells [15,19,20]. Protection elicited by vaccines expressing the RSV fusion proteins locked in their pre-fusogenic conformation (Pre-F) is actively being investigated as Pre-F stimulate greater quantities of neutralizing antibodies and total anti-G protein-specific antibodies than post-fusion form in mice, regardless of their previous exposure to RSV [21]. In our previous study, we investigated the efficacy of VLPs expressing the RSV G protein with tandem repeats (Gt) [22]. Co-displaying this antigen with Pre-F provided superior protection against RSV infection than VLPs displaying either antigen alone [23]. Although this combinatorial approach to developing an RSV vaccine was demonstrated to be effective in mice, the differences in lung virus titer and pulmonary inflammation between Pre-F and Pre-F+Gt immunization groups were negligible. Interestingly, one in silico study revealed that the steric hindrance imposed by the RSV G protein hampers neutralizing antibody binding to RSV F protein [24]. Because the Gt antigen used in our previous study is significantly larger than the conventional RSV G antigen as tandem repeats were introduced, even more steric masking effect may have been elicited to dampen the vaccine efficacy. To address this limitation, we replaced the Gt antigen with the RSV G antigen to minimize the steric hindrance. Currently, only a handful of studies have investigated the efficacy of VLPs displaying Pre-F and G. Virus-neutralizing antibodies in mice that were previously infected with RSV can be rapidly activated by VLPs co-expressing Pre-F and G proteins but not by those expressing the post-fusion conformation [25]. VLPs co-expressing both F and G antigens induced virus-specific antibodies that contributed to protection and also limited the inflammation [26]. Immunizing cotton rat dams with VLPs expressing the RSV Pre-F and G antigens enabled the transmission of neutralizing antibodies to their offspring and ensured that they were well-protected [27]. The co-expression of both antigens was also reported to be crucial for mounting enhanced immune response to the RSV F glycoprotein [28]. Yet, a large number of these aforementioned studies were mostly focused on assessing the virus-neutralizing antibody responses, and immune correlates associated with protection remain unexplored. In this study, we investigated and compared the efficacy of VLP vaccines containing Pre-F, unmodified G protein, or both and evaluated some of the immunological parameters associated with protection in mice.

## 2. Materials and Methods

### 2.1. Ethics Statement

All in vivo experimental protocols were approved by the Kyung Hee University Institutional Animal Care and Use Committee (IACUC; permit number: KHSASP-21-340). Mice were given free access to food and water ad libitum in an institution-approved housing facility with 12 h day and night cycles. Animals were monitored daily, and any mice that displayed signs of distress or suffering were humanely euthanized via CO_2_ instillation. For this study, the humane intervention point was set as weight loss exceeding 20%, and mice reaching this endpoint were placed in CO_2_ chamber with a CO_2_ flow rate of 3 L/min. 

### 2.2. Cells, Antibodies, and Viruses

*Spodoptera frugiperda* (Sf9) insect cells (Invitrogen, Waltham, MA, USA) were cultured in suspension using spinner flasks and SF900II medium (Invitrogen, Carlsbad, CA, USA) at 27 °C for the generation of recombinant baculovirus (rBV) and VLPs. HEp-2 cells were grown in tissue culture flasks using Dulbecco’s modified Eagle medium (DMEM) with 10% fetal bovine serum (FBS), penicillin and streptomycin at 37 °C with 5% CO_2_. For polyclonal anti-RSV antibody acquisition, mice were infected thrice with RSV A2 virus at 4-week intervals. One week after the final infection, sera were collected via retro-orbital plexus puncture. Monoclonal anti-RSV fusion antibody (131-2A) was purchased from Millipore and used in virus plaque assay and Western blot. The mouse monoclonal influenza A virus anti-M1 antibody was purchased from Abcam (Cambridge, UK) and used in Western blot. Horseradish peroxidase (HRP)-conjugated secondary mouse IgG antibodies were purchased from Southern Biotech (Birmingham, AL, USA). Fluorophore-conjugated antibodies were purchased from BD Bioscience (Franklin Lakes, NJ, USA) and used to perform flow cytometry. RSV A2 was originally kindly provided by Dr. Marty Moore of Emory University. RSV A2 and FI-RSV were prepared following the method previously described [8,22,29,30].

### 2.3. Generation of rBV and VLPs

A codon-optimized construct of the near full-length Pre-F antigen previously described by Patel et al. [31] was purchased from GenScript (Piscataway, NJ, USA). The influenza matrix protein 1 (M1; GenBank EF467824) cloned in pFastBac vector was prepared as described previously [32]. Upon subsequent cloning into DH10Bac competent cells, bacmid DNA was acquired from colonies (Favorgen, Cheshire, UK). Pre-F, G, or influenza M1-expressing recombinant baculoviruses (rBVs) were prepared as previously described [32,33,34]. VLPs expressing Pre-F, G, or both on the surface of influenza M1 were produced using Sf9 cells as previously described [33,35]. Briefly, Sf9 cells were transfected with rBVs expressing the RSV antigens and influenza M1 for VLP assembly. At 3 days post-infection (dpi), supernatants from the Sf9 cell culture were carefully collected by centrifuging at 4800× *g*, 30 min for 4 °C. VLPs were subjected to ultracentrifugation and purified using a sucrose density gradient. Purified VLPs were resuspended in PBS and stored at 4 °C until use as described elsewhere [33,34].

### 2.4. Characterization of RSV VLPs

Purified VLPs morphologies were identified using transmission electron microscopy (TEM). VLPs were negatively stained with 2% uranyl acetate on the sample grids and observed using the Bio-High voltage EM system (JEM-1400 Plus at 120 kV and JEM-1000BEF at 1000 kV, JEOL Ltd., Tokyo, Japan) at the Korea Basic Science Institute. Western blotting was performed to confirm the expression of pre-fusion, glycoprotein, and M1 proteins. Protein concentrations were quantified using the Micro BCA protein assay kit (ThermoFisher, Waltham, MA, USA). Briefly, proteins were loaded onto 10% sodium dodecyl sulfate-polyacrylamide gels and transferred onto a PVDF membrane (Millipore, MA, USA). Membranes were blocked with 5% skim milk prepared in Tris-buffered saline with 0.1% Tween-20 (TBST) at RT for 30 min. Blocked membranes were incubated with monoclonal anti-RSV fusion antibody (1:5000 dilution), polyclonal anti-RSV antibody from RSV-infected mice (1:1000 dilution), or monoclonal anti-M1 (1:2000 dilution) overnight at 4 °C to detect pre-fusion, glycoprotein, or M1 antigens, respectively. After washing, membranes were probed with HRP-conjugated mouse IgG antibodies (1:5000 dilution) at RT for 1 h. Bands and images were acquired using the ChemiDoc imaging system (Bio-Rad, Hercules, CA, USA).

### 2.5. Immunization and Challenge Infection

A total of 42 six-week-old female Balb/c mice were subdivided into 7 groups (n = 6 per group). Mice were grouped as follows: naïve, naïve challenge, Pre-F VLP, G VLP, Pre-F+G VLP, FI-RSV, and live RSV immunization group. For the VLP groups, mice were intranasally immunized twice at 4-week intervals with 120 µg of respective VLPs. For FI-RSV and live RSV immunization groups, mice were intranasally immunized once with FI-RSV A2 (50 µL/mouse) or live RSV A2 (1 × 10^5^ pfu/mouse), respectively. Equal volumes of inoculum were administered into each nostril using a pipette. Sera were collected 3 weeks after each immunization via a retro-orbital plexus puncture. Four weeks after the last immunization, mice were challenged with RSV A2 (4 × 10^6^ pfu/mouse). Mice were lightly anesthetized with isoflurane prior to immunization, challenge infection, and serum collection. At 5 dpi, all mice from each group were sacrificed for organ sampling and ex vivo studies.

### 2.6. Virus-Specific Antibody Responses

RSV-specific antibody responses were observed using the enzyme-linked immunosorbent assay (ELISA) as previously described [22]. Briefly, FI-RSV antigens were coated in 96-well immunoplates at a concentration of 4 µg/mL in carbonate/bicarbonate coating buffer (pH 9.6). Plates were blocked with 0.2% gelatin at 37 °C, 1 h. After washing, sera (1:100 dilution in PBST) and homogenized lung extracts (1:10 dilution in PBST) were inoculated into respective wells as primary antibodies, and plates were incubated at 37 °C for 2 h. Horseradish-peroxidase (HRP)-conjugated anti-mouse IgA, IgG, IgG1, IgG2a, and IgG2b secondary antibodies (1:2000 dilution in PBST) were added to respective wells, and plates were incubated at 37 °C, 1 h. O-phenylenediamine (OPD) dissolved in substrate buffer with 30% H_2_O_2_ was added for color development and stopped with 2N H_2_SO_4_. The aborsorbance values at 450 nm/490 nm were measured using a microplate reader.

### 2.7. Flow Cytometry and Cytokine Assays

Single cells isolated from the lungs were used to assess CD4^+^ T cell, CD8^+^ T cell, and eosinophil populations via flow cytometry as previously described [22]. Cells were stimulated with a mixture containing equal concentrations of RSV fusion and glycoprotein (Sino Biological, Beijing, China) at a final concentration of 2 µg/mL for 5 h at 37 °C. Cells were subsequently stained with fluorophore-conjugated CD3, CD4, CD8, CD11b, CD125, Siglec F, and IL-4 antibodies (BD Biosciences, Franklin Lakes, NJ, USA). Antibody-stained cells were analyzed using a BD Accuri C6 Flow Cytometer (BD Biosciences, Franklin Lakes, NJ, USA). For cytokine assay, spleens were homogenized, and isolated splenocytes were cultured in RPMI-1640 media (Lonza, Switzerland) with 2 µg/mL of the RSV fusion and glycoprotein at 37 °C for 6 days. Culture supernatants were carefully collected, and the expressions of the cytokines IL-4, IL-5, and IFN-γ were assessed using BD OptEIA ELISA kits (BD Biosciences, Franklin Lakes, NJ, USA).

### 2.8. Pulmonary Histopathology

At day 5 post-challenge, individual left lung lobes harvested from mice were fixed with 10% neutral buffered formalin. The lung tissues (n = 3) were embedded in paraffin after dehydration, sectioned, and stained with either hematoxylin and eosin (H&E) or periodic acid–Schiff (PAS). Pathological changes were evaluated based on the degree of leukocyte aggregation, presence of mucus, etc., and scored on a scale of 0 to 5 (0 = normal naïve parameters; 5 = severe symptom). At least 9 sections per group were obtained for histopathologic analysis.

### 2.9. Neutralizing Activity Analysis and Virus Titer

Sera collected after the final immunization were inactivated at 56 °C for 30 min and serially diluted for serum virus neutralization assay. Diluted sera were mixed with live RSV A2 viruses and incubated at 37 °C for 1 h. Confluent monolayers of HEp-2 cells cultured in 24-well plates were briefly washed and incubated with sera–virus mixtures at 37 °C for 1 h. After aspirating the sera–virus mixtures from the wells, plates were overlayed with 1 mL noble agar and incubated at 37 °C with 5% CO_2_ for 2 days. The agar overlay was gently removed, and cells were fixed with ice-cold fixatives comprising equal volumes of acetone and methanol for 10 min at RT. After washing off excess fixatives, the plates were blocked with 5% skim milk in PBS for 30 min at RT. Monoclonal anti-RSV fusion antibody (1:2000 dilution in PBS) and HRP-conjugated anti-mouse IgG antibody (1:2000 dilution in PBS) were used as primary and secondary antibodies, respectively. Diluted antibodies were added to each well (150 µL/well) and incubated at 37 °C for 1 h. Then, 3,3′-diaminobenzidine (DAB) substrate was used to develop the plaques. Viral titers in the lung were quantified on day 5 post-challenge by plaque assay. Lung tissues collected from mice were individually homogenized with 1 mL of PBS PBS using a syringe. The lung homogenates were filtered through cell strainers (100 µm pore size) and centrifuged at 2000 rpm for 10 min to collect supernatants. Supernatants were used to determine the lung virus titer using the plaque assay method described above.

### 2.10. Statistical Analysis

All parameters were recorded for individual mice from each group. Data were presented as mean ± SEM. Statistical significance between the groups was determined by performing a one-way analysis of variance (ANOVA) with Tukey’s post hoc test and a two-tailed Student’s *t-*test using GraphPad Prism 5 software (San Diego, CA, USA). Differences between the groups were considered to be statistically significant for *p* < 0.05.

## 3. Results

### 3.1. Immunoblotting and Electron Microscopy Characterization of VLPs

**Figure 1:**
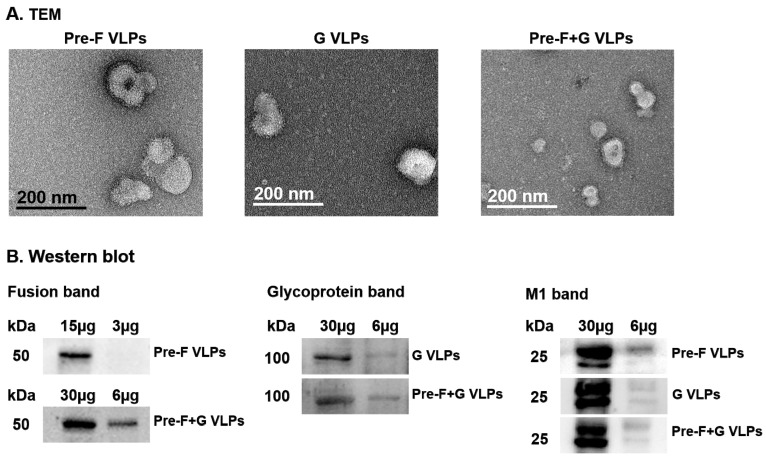
Characterization of the VLPs. VLPs expressing the RSV Pre-F, G, or both Pre-F and G antigens were visualized by transmission electron microscopy after negative staining (**A**). Influenza M1, RSV fusion, and glycoprotein antigen expressions on the VLPs were determined by Western blots (**B**).

The morphology of VLP vaccines (Pre-F VLPs, G VLPs, Pre-F+G VLPs) was determined by electron microscopy (Figure 1A). The VLP vaccines exhibited irregular spherical shapes under the influence of the influenza M1 core protein, and sharp spikes were observed on the surface. Western blot was performed to identify the components of the VLPs (Figure 1B). Pre-fusion (50 kDa), G glycoprotein (70 kDa), and M1 (28 kDa) proteins were probed with monoclonal anti-RSV fusion antibody, polyclonal anti-RSV antibody obtained from RSV-infected mice, and monoclonal anti-M1 antibody, respectively.

### 3.2. Antibody and Neutralization Responses in Serum

**Figure 2:**
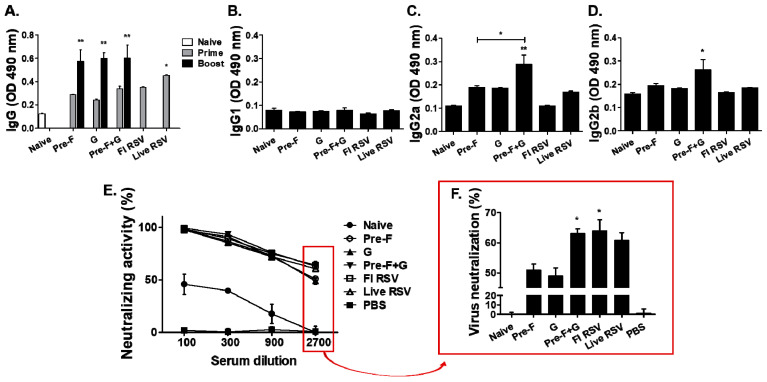
Serum antibody responses and neutralizing activity. RSV A2-specific IgG (**A**) antibody response was determined in the sera (n = 6) at week 3 after prime (gray bars) and boost (black bars) immunization. RSV A2-specific IgG1 (**B**), IgG2a (**C**), and IgG2b (**D**) antibody responses were observed in the sera (n = 6) at week 3 after boost immunization. A higher level of IgG antibody response was determined in immunized mice compared to naïve mice ((**A**), * *p* < 0.05, ** *p* < 0.01). A higher level of IgG2a antibody responses was determined in Pre-F+G VLPs immunized mice compared to naïve, FI-RSV, and Live RSV groups ((**C**), ** *p* < 0.01, * *p* < 0.05). In addition, a higher level of IgG2b antibody response was detected in the Pre-F+G VLPs group compared to the naïve group ((**D**), * *p* < 0.05). A dose-dependent relationship between serum dilution and virus neutralization activity was observed (**E**). Neutralizing antibody response exceeding 50% virus neutralization was detected until 2700-fold serum dilution, and Pre-F+G VLP-immunized mice showed significantly higher neutralizing responses than Pre-F VLPs immunized mice ((**F**), * *p* < 0.05).

RSV-specific serum antibody responses were determined in mice as shown in Figure 2A–D. High levels of IgG antibody responses were observed in immunized mice (Figure 2A), especially after the boost immunization with a significantly higher level of IgG antibody response being elicited by VLP immunization groups (Pre-F, G, and Pre-F+G) compared to the naïve group (* *p* < 0.05, ** *p* < 0.01). IgG subclasses IgG1, IgG2a, and IgG2b antibody responses were assessed after boost immunization (Figure 2B–D). Overall, low IgG1 responses were observed (Figure 2B). In contrast, significant increases in IgG2a and IgG2b responses were detected from the sera of Pre-F+G VLP-immunized mice compared to control mice (Naive, FI RSV, Live RSV; Figure 2C,D, * *p* < 0.05, ** *p* < 0.01). To confirm that the neutralizing antibody was successfully induced by the VLP vaccines after boost immunization, viral titers were measured in vitro (Figure 2E,F). As shown in Figure 2F, high levels of virus-neutralizing antibody responses were observed from the sera of immunized mice compared to naïve sera. Significant differences were only observed in Pre-F+G VLP (63%) and FI-RSV (63.8%) groups in comparison to naïve control, while Pre-F VLP immune sera only elicited roughly 50% virus neutralization (Figure 2F, * *p* < 0.05).

### 3.3. VLP Immunization Induced Antibody Response in the Lungs and Splenic Cytokine Responses

**Figure 3:**
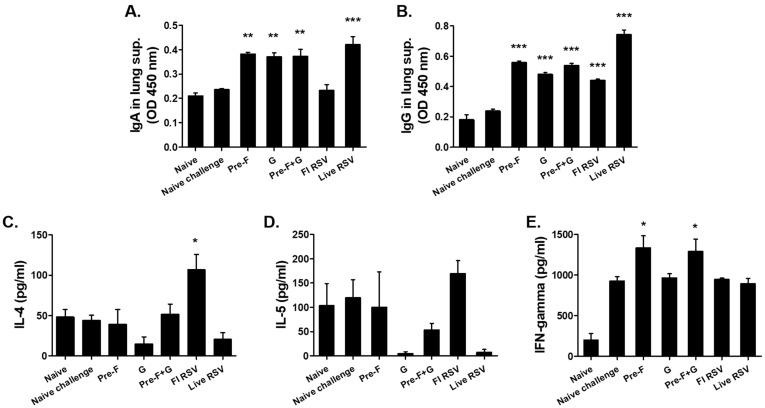
Antibody responses in the lung and cytokine responses in the spleen. Respiratory syncytial virus (RSV) A2-specific IgA (**A**) and IgG (**B**) responses were detected from lung extracts of mice collected at day 5 post-challenge. Significantly higher levels of IgA and IgG responses were observed in immunized mice than naïve challenged mice ((**A**,**B**); ** *p* < 0.01, *** *p* < 0.001). Splenic cytokines were detected by culturing splenocytes with formalin-inactivated RSV (2 µg/mL) for 6 days. IL-4 (**C**), IL-5 (**D**), and IFN-γ (**E**) production were measured and the mean values were compared to control groups (* *p* < 0.05).

Antibody responses were evaluated using the lung homogenate supernatants, which were collected after the challenge infection (Figure 3A,B). High levels of IgA and IgG antibody responses were detected in immunized groups compared to the naïve challenged group (** *p* < 0.01, *** *p* < 0.001). The highest IL-4 induction was observed in the FI-RSV group, which was significantly greater compared to all other groups (Figure 3C). On the contrary, no significant changes to the production of Th2 cytokine IL-5 were detected (Figure 3D). Considering that IgG2a and IgG2b were increased in the serum antibody response, it was expected that the Th1 response would increase and the related cytokines would be detected at high levels. As expected, induction of the Th1 cytokine IFN-γ increased in all groups except for the naïve group, with significant increases being detected from Pre-F and Pre-F+G VLP groups (Figure 3E; * *p* < 0.05).

### 3.4. VLP Immunization Reduced Eosinophil and IL-4-Producing CD4^+^ T Cell Counts

**Figure 4:**
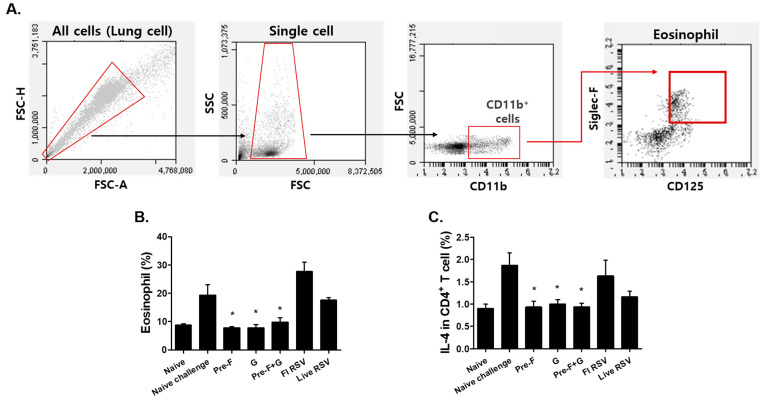
Eosinophil and IL-4 producing CD4^+^ T cell counts. To isolate the eosinophil and IL-4-producing CD4^+^ T cell phenotypes in the lung, lung cells were stained with fluorescent markers (CD11b, CD125, Siglec-F, CD3, CD4, and IL-4) and analyzed by BD Accuri C6 software (**A**–**C**). VLP-immunized groups showed significantly lower levels of eosinophil compared to naïve challenge ((**B**), * *p* < 0.05). A significantly lower level of IL-4-producing CD4^+^ T cells was observed in VLP-immunized mice than naïve challenged mice ((**C**), * *p* < 0.05).

RSV infection is frequently accompanied by the influx of eosinophils into the lungs, and assessing the eosinophil population is important to accurately determine a vaccine’s protective efficacy. To evaluate this, lung cells were stained with CD11b, CD125, and Siglec-F antibodies and analyzed by flow cytometry (Figure 4A). In contrast to the high levels of eosinophils shown in naïve challenge and FI-RSV immunized groups, eosinophil frequencies were significantly lower in VLP-immunized groups (Figure 4B; * *p* < 0.05). Confirming the increase in IL-4-producing CD4^+^ T cells can infer whether the cellular response is an allergic or hypersensitivity reaction. Similar to the eosinophil response, VLP-immunized groups showed a significantly lower population of IL-4-producing CD4^+^ T cells than the naïve challenge or FI-RSV groups (* *p* < 0.05; Figure 4C).

### 3.5. Pre-F+G VLP Immunization Induced Cellular Immune Responses

**Figure 5:**
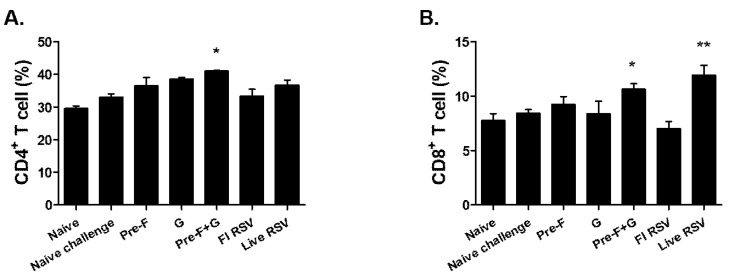
CD4^+^ and CD8^+^ T cell counts in lung. To isolate the CD4^+^ and CD8^+^ T cells in the lung, lung cells were stained with surface markers (CD3, CD4, and CD8) and analyzed with BD Accuri software (**A**,**B**). Data were acquired on an individual basis from each group (n = 6) and presented as percentages among the CD3^+^ T cells. Pre-F+G VLP-immunized mice showed higher levels of CD4^+^ and CD8^+^ T cells compared to naïve control mice (* *p* < 0.05, ** *p* < 0.01).

To determine the cellular immune responses, lung cells stimulated with RSV fusion and G glycoproteins were stained with CD3, CD4, and CD8 antibodies. Significant changes in the means of CD4^+^ T cell population between the groups were not detected except for PreF+G VLP immunized mice (Figure 5A; * *p* < 0.05). Similar to CD4^+^ T cell responses, CD8^+^ T cell inductions were significantly enhanced by Pre-F+G VLP immunization. Live RSV immunization also resulted in substantial CD8^+^ T cell influx compared to the naïve control group (Figure 5B; * *p* < 0.05, ** *p* < 0.01). Although marginal increases in T cell populations were demonstrated by Pre-F VLPs, the changes were not statistically significant.

### 3.6. VLP Immunization Reduced Lung Inflammation

**Figure 6:**
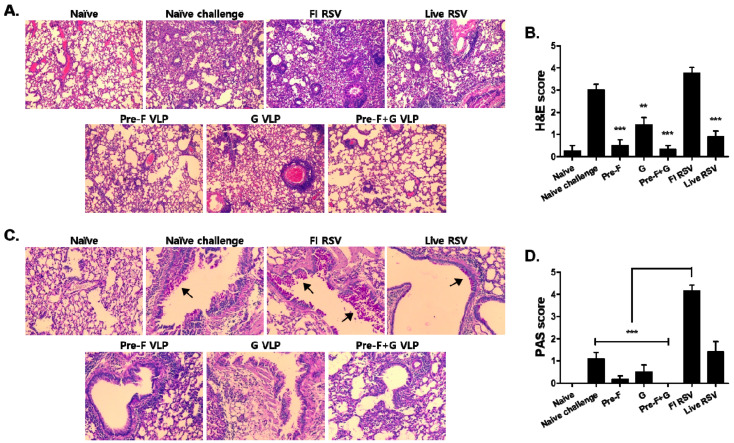
Lung pathology assessment. Lung tissues (n = 3) were collected from individual mice at day 5 post-challenge, and the micro-sectioned tissues were stained with hematoxylin and eosin (H&E) and periodic acid–Schiff (PAS) to observe pathological changes in the lungs. H&E staining (**A**) and H&E pathology scores (**B**) for inflammation were determined. PAS staining (**C**) and its corresponding score (**D**) for mucin production were determined. Magenta-stained areas show mucin production and these were indicated using arrows. VLP-immunized mice showed lower cellular influx compared to naïve challenge ((**B**), ** *p* < 0.01, *** *p* < 0.001). VLP-immunized mice showed significantly lower PAS scores than the FI-RSV group ((**D**), *** *p* < 0.001). All images were acquired at 200× magnification.

Pulmonary histopathology examinations revealed that VLP immunization alleviates inflammation induced by RSV infection. To determine lung histopathology, H&E staining was performed on lung tissue, and the severity of inflammatory response was quantified by scoring (Figure 6A,B). Compared to the naïve, naïve challenge and the FI-RSV control groups had high histopathology scores ranging between 3 and 5 due to excessive cellular influx. On the other hand, the VLP immunization groups showed significantly less cellular influx compared to the naïve challenge. The histopathology score for the Pre-F+G VLP immunization group was 0–0.5, which resembled those of naïve control (Figure 6B; ** *p* < 0.01, *** *p* < 0.001). An excessive secretion of airway mucus causes airway obstruction, which is one of the main symptoms of severe RSV disease. PAS-stained lung tissue was used to determine mucus production in the lungs (Figure 6C). Excessive mucus production was observed in the FI-RSV group, with the highest PAS score in the 4–5 range. The PAS scores of VLP-immunized groups were considerably lower than the controls, with the Pre-F+G VLP group receiving the lowest histopathology score (Figure 6D; *** *p* < 0.001).

### 3.7. VLP Immunization Reduced Lung Viral Titers

**Figure 7:**
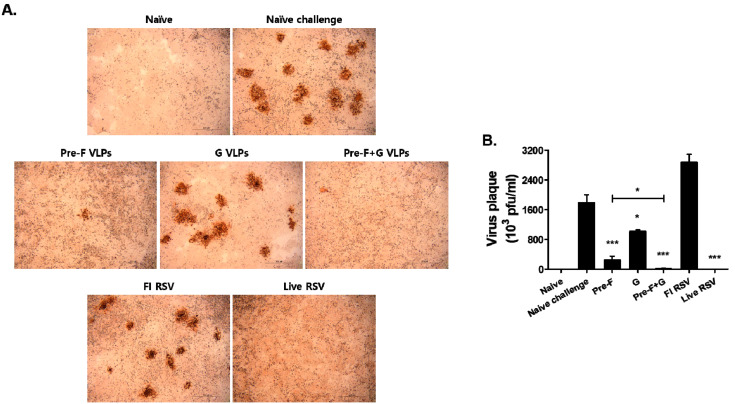
Lung viral titer after RSV A2 infection. Lung viral titers were determined individually (n = 6) from the lung homogenate. Lung tissues were collected from individual mice 5 days after RSV infection. To confirm the viral plaque formation, DAB substrate was used ((**A**), scale bar, 500 μm). VLPs immunized mice showed significantly lower levels of viral titers than naïve challenged mice ((**B**), * *p* < 0.05, *** *p* < 0.001).

Viral titers in the lungs were assessed after RSV infection to evaluate vaccine efficacy. As shown in Figure 7A, the viruses in the lung homogenates of infected mice were used to infect HEp-2 cells, and plaque formation ensued within a few days. Plaque quantification results revealed that significantly lower virus titers were detected in VLP-immunized mice compared to naïve challenge (Figure 7B; * *p* < 0.05, *** *p* < 0.001). Of the three VLP groups, the lowest virus titer was observed in homogenates of the Pre-F+G VLP group. Viral loads were also significantly diminished for Pre-F VLP, with G VLP immunization resulting in the weakest viral titer reduction (* *p* < 0.05).

## 4. Discussion

The vaccine-induced exacerbation of respiratory disease and exuberant inflammatory responses are some of the features associated with RSV infection barring vaccine development. While the alleviation of such symptoms can be an important indicator of vaccine efficacy, an effective antiviral drug or vaccine for acute RSV infection remains unavailable [36]. Although numerous VLP vaccine studies involving F and G proteins were conducted, a large portion of these studies was based on the post-fusogenic form of the F protein. To date, only a few studies evaluated the efficacy of VLPs expressing both RSV Pre-F and G protein. In one study, Cullen et al. [21] demonstrated that the conformation of the RSV F protein has a profound effect on eliciting neutralizing antibodies in RSV-primed mice. The identical group of authors also revealed the superior protection conferred by VLPs expressing the Pre-F antigen compared to Post-F or subsequent RSV challenge infection in RSV-primed animals [25]. The developmental potential of these VLP vaccines was also confirmed using maternal immunization models [37,38]. Despite these promising data from in-depth analyses, a large bulk of these aforementioned studies were predominantly focused on the humoral immunity associated with the VLPs, especially the neutralizing antibody responses. To further advance our understanding, we chose to investigate cellular immunity and other immune correlates, as relatively less attention was paid to these aspects. Our findings revealed that mice immunized with Pre-F+G VLPs provided better protection than Pre-F VLPs or G VLPs-immunized mice, as implicated by the T cell influx and diminished eosinophilia in the lungs. Specifically, our data showed that high levels of cellular influx and mucin production in FI-RSV immunized mice resulted in severe inflammatory response upon RSV infection. In contrast, pulmonary inflammation symptoms in VLP-immunized mice occurred to a lesser degree compared to the controls.

Recently, the importance of RSV G protein with respect to F protein conformational stability and immunogenicity was reported. The G antigens expressed on the VLPs significantly contributed to the induction of robust neutralizing antibody titers and stabilized the Pre-F conformation of the fusion protein, all of which contributed to better protection in cotton rats compared to Pre-F VLPs alone [28]. Consistent with this finding, Pre-F+G VLPs in our study also conferred protection that partly exceeded those induced by the Pre-F or G VLPs. As aforementioned, in our previous study, the differences in lung virus titers from Pre-F and Pre-F+Gt VLPs were negligible [23]. As we anticipated, replacing the Gt antigen with the conventional RSV G enhanced protection. In support of this notion, Pre-F+G VLPs demonstrated near-complete virus clearance, which was significantly different from those demonstrated by the Pre-F VLPs. Furthermore, immunizing mice with the Pre-F+G VLP prevented mucin production, which is reported to exacerbate RSV-associated lung pathology when produced in excessive quantities [39,40]. Based on our findings, the PreF+G VLPs were highly efficacious and suppressed the development of abnormal inflammatory responses. Although several immunological parameters between the two groups were similar, the findings presented here further support the developmental potential of the VLPs co-expressing Pre-F and G antigens.

Substantial CD4 and CD8 T cell influx were observed in the lungs of naïve mice. This was to be expected, as evidenced by the presence of circulating naïve T cells that enter the non-lymphoid organs [41,42]. The failure of the FI-RSV vaccine is largely due to the induction of an excessive inflammatory response upon RSV re-infection [8,43]. IL-4-producing CD4^+^ T cell induction following antigen-dependent T cell priming can significantly influence the development of airway inflammation and hypersensitivity and this, combined with activated inflammatory cells and eosinophils, can cause complex pathologies in an autocrine manner [44,45]. Compared to naïve challenge (2.3%) or the FI-RSV (3.7%) group upon challenge infection, the Pre-F, G, and Pre-F+G VLPs we generated showed significantly reduced eosinophilia in the lungs by 0.5%, 0.6%, and 0.7%, each respectively. IL-4-producing CD4^+^ T cell inductions, as well as the lung histopathology results (Figure 6), also showed a similar pattern to that of eosinophils. These results support the theory that IL-4 induction by CD4^+^ T cells has a significant effect on the development of airway inflammation. It is widely accepted that Th2-skewed immunity is associated with VED formation, as is the case with FI-RSV vaccines [46,47]. However, shifting the host’s immunity to a more Th1-dominant response via VLP immunization was reported to suppress VED [48]. In line with this notion, Pre-F+G VLP immunization elicited substantially fewer Th2 cytokines IL-4 and IL-5 compared to the naïve-challenge or the FI-RSV control groups whilst promoting the production of the Th1 cytokine IFN-γ. The shift toward Th1-biased immunity demonstrated by Pre-F and Pre-F+G VLPs is thought to be the major contributor to protection.

The stimulation of mucosal and humoral immunity is important for providing protection against respiratory infections [49]. In order to confirm the presence of vaccine-induced mucosal and humoral immunity that can mitigate RSV infection, VLPs were intranasally inoculated, and antibody responses were determined. Mice immunized with the VLPs showed significantly higher levels of RSV-specific serum IgG and lung IgA compared to the FI-RSV group. In our previous study, we could not determine whether the vaccine we generated induced either Th1 or Th2 responses because we did not assess the IgG subclasses or cytokines associated with the Th1/Th2 immune responses [23]. Here, to address the limitations of our previous works, the level of IgG subclasses in sera was measured to determine Th1/Th2 responses. Th1 immune response is mainly induced upon intracellular pathogen infection and generally produces IFN-γ, IL-2, TNF-β, etc., which are critical for macrophage activation and eliciting cell-mediated immunity. In contrast, the Th2 cells produce IL-4, IL-5, IL-10, and IL-13 and are responsible for the eosinophil activity and inhibition of several macrophage functions [50]. Our serum ELISA results indicate that mice immunized with Pre-F+G VLPs elicited high levels of IgG2a and IgG2b responses, which are strongly associated with Th1 immunity. This is consistent with high levels of IFN-γ production in the spleens of mice post-challenge infection. In particular, it is thought that mice immunized with Pre-F+G VLPs induced a stronger Th1 response than other groups. In addition, VLP-immunized mice showed a high level of neutralizing antibody response, with Pre-F+G VLPs inducing greater virus-neutralizing activity than Pre-F VLPs.

One clinical study reported that during acute RSV infection, the concentration of antibodies directed toward the Pre-F in the sera of infants is 3-fold and 30-fold greater than those directed against post-F and G antigens, respectively. The identical study also revealed that Pre-F-specific antibodies demonstrated the highest virus-neutralizing activity [51]. In our study, the highest neutralization titer was observed from Pre-F+G and FI-RSV immunization groups. Similar findings were reported by several research groups. For example, it was recently reported that immunizing cotton rats with viral-vectored vaccines co-expressing both F and G antigens elicited a stronger virus-neutralizing response than single antigen vaccines [52]. Moreover, VLPs expressing both Pre-F and G ectodomains elicited substantially enhanced neutralizing antibody titers than those solely expressing either Pre-F or G alone [28]. In our study, the high virus-neutralizing antibody titer elicited by FI-RSV immunization, hardly any lung titer reductions were observed. Consistent with this finding, in our previous studies, FI-RSV immunization elicited neutrazing antibody response without plaque reduction in the lungs of mice [23,53]. Contrastingly, one research group reported that FI-RSV immunization provided potent serum-mediated virus neutralization and inhibited RSV replication in the lungs of mice [54]. Another study revealed that FI-RSV failed to elicit robust neutralizing antibody titers, which consequently led to high lung virus titers in mice [15]. While we do not know the definitive cause of this discrepancy between research groups, we speculate that FI-RSV preparation, immunization regimen, dosage, choice of animal model, and other experimental factors likely contributed to such outcomes. Nonetheless, all of the research seems to agree that FI-RSV immunization induces severe pulmonary histopathology, as shown here.

## 5. Conclusions

Collectively, Pre-F+G VLP immunization induced high titers of neutralizing antibodies, decreased pulmonary inflammation, reduced eosinophil influx, and enhanced IL-4-producing CD4^+^ T cells, all of which contributed to inhibiting viral replication in the lungs of mice. Although noticeable differences in lung titers were observed, all of the VLP-immunization groups significantly lowered the lung viral titers compared to naïve challenge mice, thereby indicating that these VLP vaccines are capable of responding to RSV infection. When comparing Pre-F VLPs and G VLPs, protective efficacy was superior in the former of the two with VLPs co-expressing both antigens inducing the best protection. In our study, Pre-F was the major antigen involved in protection, while G glycoprotein played an auxiliary role. These study results suggest that the VLPs vaccine expressing Pre-F and G antigens provides better protection than VLPs expressing either antigen alone and has sufficient potential as an RSV vaccine to be developed in the future.

## Data Availability

The original contributions presented in this study are included in the article. Further inquiries can be directed to the corresponding author.
